# Tensor-valued diffusion MRI differentiates cortex and white matter in malformations of cortical development associated with epilepsy

**DOI:** 10.1111/epi.16605

**Published:** 2020-07-15

**Authors:** Björn Lampinen, Ariadne Zampeli, Isabella M. Björkman-Burtscher, Filip Szczepankiewicz, Kristina Källén, Maria Compagno Strandberg, Markus Nilsson

**Affiliations:** 1 Clinical Sciences Lund, Medical Radiation Physics, Lund University, Lund, Sweden; 2 Clinical Sciences Lund, Neurology, Lund University, Lund, Sweden; 3 Department of Radiology, Sahlgrenska Academy, University of Gothenburg, Gothenburg, Sweden; 4 Clinical Sciences Lund, Diagnostic Radiology, Lund University, Lund, Sweden; 5 Brigham and Women’s Hospital, Harvard Medical School, Boston, Massachusetts; 6 Clinical Sciences Lund, Department of Clinical Sciences Helsingborg, Lund University, Lund, Sweden

**Keywords:** axons, microscopic diffusion anisotropy, myelin

## Abstract

**Objective:**

Delineation of malformations of cortical development (MCD) is central in presurgical evaluation of drug-resistant epilepsy. Delineation using magnetic resonance imaging (MRI) can be ambiguous, however, because the conventional T_1_- and T_2_-weighted contrasts depend strongly on myelin for differentiation of cortical tissue and white matter. Variations in myelin content within both cortex and white matter may cause MCD findings on MRI to change size, become undetectable, or disagree with histopathology. The novel tensor-valued diffusion MRI (dMRI) technique maps microscopic diffusion anisotropy, which is sensitive to axons rather than myelin. This work investigated whether tensor-valued dMRI may improve differentiation of cortex and white matter in the delineation of MCD.

**Methods:**

Tensor-valued dMRI was performed on a 7 T MRI scanner in 13 MCD patients (age = 32 ± 13 years) featuring periventricular heterotopia, subcortical heterotopia, focal cortical dysplasia, and polymicrogyria. Data analysis yielded maps of microscopic anisotropy that were compared with T_1_-weighted and T_2_-fluid-attenuated inversion recovery images and with the fractional anisotropy from diffusion tensor imaging.

**Results:**

Maps of microscopic anisotropy revealed large white matter-like regions within MCD that were uniformly cortex-like in the conventional MRI contrasts. These regions were seen particularly in the deep white matter parts of subcortical heterotopias and near the gray-white boundaries of focal cortical dysplasias and polymicrogyrias.

**Significance:**

By being sensitive to axons rather than myelin, mapping of microscopic anisotropy may yield a more robust differentiation of cortex and white matter and improve MCD delineation in presurgical evaluation of epilepsy.

## 1 | INTRODUCTION

Malformations of cortical development (MCD) are important causes of drug-resistant epilepsy, a condition that affects millions worldwide and is associated with significant morbidity and mortality.^1^ MCD comprise a diverse group of gray matter lesions related to developmental disruption of neuronal proliferation, migration, and organization. Disrupted neuronal migration from the ventricular and subventricular zones to the cortical plate results in misplacement of cortical tissue close to the ventricles (periventricular heterotopia [PH]) or within white matter (subcortical heterotopia [SH]). Disrupted neuronal organization within the cerebral cortex manifests as abnormal cortical layering, for example, on the local level in focal cortical dysplasia (FCD) or across whole gyri in polymicrogyria (PMG).^2^ MCD may have intrinsic epileptogenic activity and produce medically refractory seizures that are most effectively treated through surgical resection.

Delineation of cortical abnormalities with magnetic resonance imaging (MRI) is central in presurgical evaluation of epilepsy and to the odds of achieving seizure freedom.^3,4^ However, the delineation of MCD can be ambiguous with conventional MRI, because the T_1_- and T_2_-weighted contrasts depend on myelin for differentiation of cortex and white matter. In the normal adult brain, a low myelin content makes cortex hypointense to white matter on T_1_-weighted images and hyperintense to white matter on T_2_-weighted images.^5^ The myelin content changes during maturation,^5–7^ however, and may be altered in pathologies such as MCD.^8,9^ Consequently, variations in myelin content within both cortex and adjacent white matter may cause MCD findings on T_1_- and T_2_-weighted images to change size, become undetectable or indicate “pseudothickening” of the cerebral cortex in disagreement with histopathology.^6,8,10^ On T_2_-weighted images, differentiation of cortex and white matter is additionally complicated by iron content, which is typically higher in gray matter and may counteract the myelin-based contrast in some cortical regions.^11–13^ Thus, a more robust delineation of MCD in presurgical evaluation of epilepsy requires an imaging contrast that can differentiate cortex and white matter based on a feature other than myelin.

Diffusion MRI (dMRI) probes tissue microstructure by the diffusive motion of water molecules. The technique is sensitive to the anisotropic diffusion induced by elongated structures such as axons.^14^ The diffusion anisotropy from axons is not strongly dependent on myelin,^15,16^ wherefore using dMRI to differentiate cortex and white matter based on axonal content may be a more robust alternative to T_1_- and T_2_-weighted contrasts. Diffusion anisotropy has historically been quantified by the fractional anisotropy (FA) from diffusion tensor imaging. However, differentiation of cortex and white matter based on the FA is ambiguous, because the FA reflects diffusion anisotropy on the macroscopic voxel level. Thus, a low FA does not necessarily reflect a low axonal content but could also reflect a lower orientation coherence of axons.^17^ The novel “tensor-valued” dMRI technique overcomes this limitation by performing diffusion encoding in multiple directions simultaneously.^18–23^ Acquiring data with different shapes of the “b-tensor” yields independent information that isolates the influence of the microscopic diffusion anisotropy. Previous studies have suggested that microscopic anisotropy can be estimated from data acquired with conventional encodings only.^24^ However, results from data acquired with multiple shapes of the b-tensor have shown such measures to be highly inaccurate.^25–28^Fortunately, tensor-valued dMRI is both fast and available on common clinical platforms (https://github.com/filip-szczepankiewicz/fwf_seq_resources).^29,30^ By providing an axon-sensitive measure through the microscopic anisotropy, tensor-valued dMRI may be a promising tool for differentiation of cortex and white matter in conditions affecting myelin such as MCD.

This study examined 13 patients with MCD on a 7 T MRI scanner using tensor-valued dMRI as well as T_1_-weighted and T_2_-fluid-attenuated inversion recovery (FLAIR) sequences. We hypothesize that mapping microscopic anisotropy with tensor-valued dMRI may improve MCD delineation by enabling differentiation of cortex and white matter based on a voxel’s axonal content rather than its myelin content.

## 2 | MATERIALS AND METHODS

### 2.1 | Patients

Patients with radiologically diagnosed MCD were recruited between 2016 and 2019 from a study of 7 T MRI in epilepsy at Skåne University Hospital’s Department of Neurology, Lund, Sweden. The inclusion criteria were nonresected lesion, lesion size covering multiple voxels in the dMRI data, lesion location not affected by signal dropout or severe echo-planar imaging (EPI) distortions, and high-resolution T_1_-weighted images available for coregistration. Of 34 eligible patients, 21 were excluded. The causes for exclusion were complete or partial lesion resection (three patients), lesion too small to be resolved in the dMRI data (three patients), lesion location affected by imaging artifacts (six patients), missing or incomplete dMRI data (eight patients), and missing T_1_-weighted images (one patient).

The resulting patient population consisted of 13 subjects, eight females and five males, with a mean ± standard deviation age of 32 ± 13 years at the date of examination. See Table S1 for an overview of patient characteristics.

The study was approved by the Swedish Ethical Review Authority, and all subjects gave written informed consent according to recommendations of the Declaration of Helsinki.

### 2.2 | MRI acquisition

Whole-brain MRI data were acquired on an actively shielded 7 T Achieva (Philips) equipped with a dual-channel transmit and 32-channel receive head coil (Nova Medical). For increased field homogeneity, dielectric pads were used during image acquisition. dMRI data were acquired using a prototype diffusion-weighted spin-echo sequence^29^ for b-values up to 2.0 ms/μm^2^ using both linear and spherical shapes of the b-tensor.^20^ The spatial resolution was 2 × 2 × 4 mm^3^. See Table S2 for a detailed description of the diffusion sequence. T_1_-weighted images were acquired using a three-dimensional (3D) turbo-field echo sequence with repetition time = 8 milliseconds, echo time = 2.8 milliseconds, flip angle = 7°, and 0.6-mm isotropic voxel dimensions. T_2_-FLAIR images were acquired using a 3D spin-echo sequence^31^ with repetition time = 6000 milliseconds, echo time = 390 milliseconds, flip angle = 55°, and a spatial resolution of 0.69 × 0.69 × 1.4 mm^3^ (reconstructed to 0.69 × 0.69 × 0.7 mm^3^). The acquisition times for the T_1_-weighted and T_2_-FLAIR sequences were 11:46 and 10:00 minutes, respectively.

### 2.3 | MRI postprocessing and analysis

The dMRI data were corrected for eddy currents and subject motion using ElastiX with extrapolated target volumes,^32^ and subsequently smoothed using a 3D Gaussian kernel with a standard deviation of 0.42 times the voxel dimensions. The q-space trajectory imaging model^23^ was fitted to the data voxel-by-voxel using linear regression with heteroscedasticity correction to yield a metric of the microscopic anisotropy (microscopic diffusion anisotropy [MK_A_])^22^ together with the conventional FA. All postprocessing and model fitting of dMRI data was performed using MATLAB (R2015b, MathWorks) together with the multidimensional dMRI toolbox,^33^ available at https://github.com/markus-nilsson/md-dmri. The T_1_-weighted and T_2_-FLAIR images were bias field-corrected using FMRIB Software Library FAST. For the purpose of visualization and region of interest (ROI) definition, the diffusion parameter maps and the T_2_-FLAIR images were coregistered to the T_1_-weighted images using ElastiX and rigid-body transformations. To improve performance, the T_1_-weighted images were downsampled to 1.5-mm isotropic voxel dimensions before coregistration.

### 2.4 | Comparison of MRI contrasts in MCD

Two approaches were used to investigate whether mapping microscopic anisotropy by the MK_A_ may improve the differentiation of cortex and white matter in the delineation of MCD. First, we compared the appearance of the MCD in the MK_A_ maps with that in the conventional MRI contrasts (T_1_-weighted, T_2_-FLAIR, and FA). Second, we compared the image intensity distributions of the MCD between the different contrasts. For each lesion and each contrast, we quantified the percentage of voxels classified as “cortex-like” or “white matter-like.” This classification utilized information drawn from normal-appearing cortex and white matter. Details of the procedure are given below.

### 2.5 | Regions of interest

As a basis for all investigations, ROIs were placed in MCD and in normal-appearing tissue. For all patients, suspected MCD were localized and delineated with ROIs in the T_1_-weighted images. The delineation aimed to include cortical abnormalities but to exclude associated white matter, even if suspected pathological. Thus, with the exception of blurred gray-white boundaries in FCD, only tissue that was isointense with cortex was included. For very large lesions, including some PMG and SH, the ROIs were limited to representative parts. For one PMG patient, three ROIs subdivided one large lesion into three separate parts that engaged different gyri and featured different morphological characteristics. The reviewing was performed by B.L. together with I.M.B.-B., a senior neuroradiologist. Normal brain tissue was defined on the T_1_-weighted images by placing ROIs in normal-appearing cortex and its adjacent white matter on the contralateral side to each lesion in each patient.

All ROI definition was performed in the coregistered T_1_-weighted downsampled image space. However, the dMRI data featured some geometric distortions from the EPI readout. To minimize the influence of partial volume effects from such distortions, as well as from the lower native resolution of the dMRI data, the lesion ROIs were drawn with a margin of one to two voxels. Furthermore, each ROI was carefully inspected in the dMRI data using the nondiffusion-weighted images. Voxels with cerebrospinal fluid contamination were removed and, when necessary, the dMRI images were manually translated in 3D space.

### 2.6 | Intensity distributions and differentiation of cortex and white matter

Intensity distributions were extracted from each ROI in each contrast. Average distributions were obtained for normal brain tissue and for each of the different MCD types by averaging the distributions between ROIs.

Optimal intensity thresholds for differentiation of cortex and white matter were obtained for each contrast through a receiver operating characteristic (ROC) analysis on the average distributions from normal brain tissue. Using these thresholds, contrast-specific classifications were obtained for each lesion voxel as either cortex-like or white matter-like.

The T_1_-weighted and T_2_-FLAIR intensities were normalized, for each ROI, by division with the mean intensity within a reference ROI in the same subject. For T_1_-weighted intensities, the lesion ROIs were referenced to the adjacent normal-appearing white matter and the normal brain tissue ROIs were referenced to the normal white matter ROI (corresponding to each location). For T_2_-FLAIR intensities, all ROIs were referenced to the relatively homogeneous anterior corona radiata.

### 2.7 | Data accessibility

The data that support the findings of this study—metadata, thumbnail images, and extracted parameter values—are available from the corresponding author upon request.

### 2.8 | Statistical methods

The ROC analysis was performed with the *perfcurve* function in MATLAB.

## 3 | RESULTS

### 3.1 | MCD localization

**FIGURE 1:**
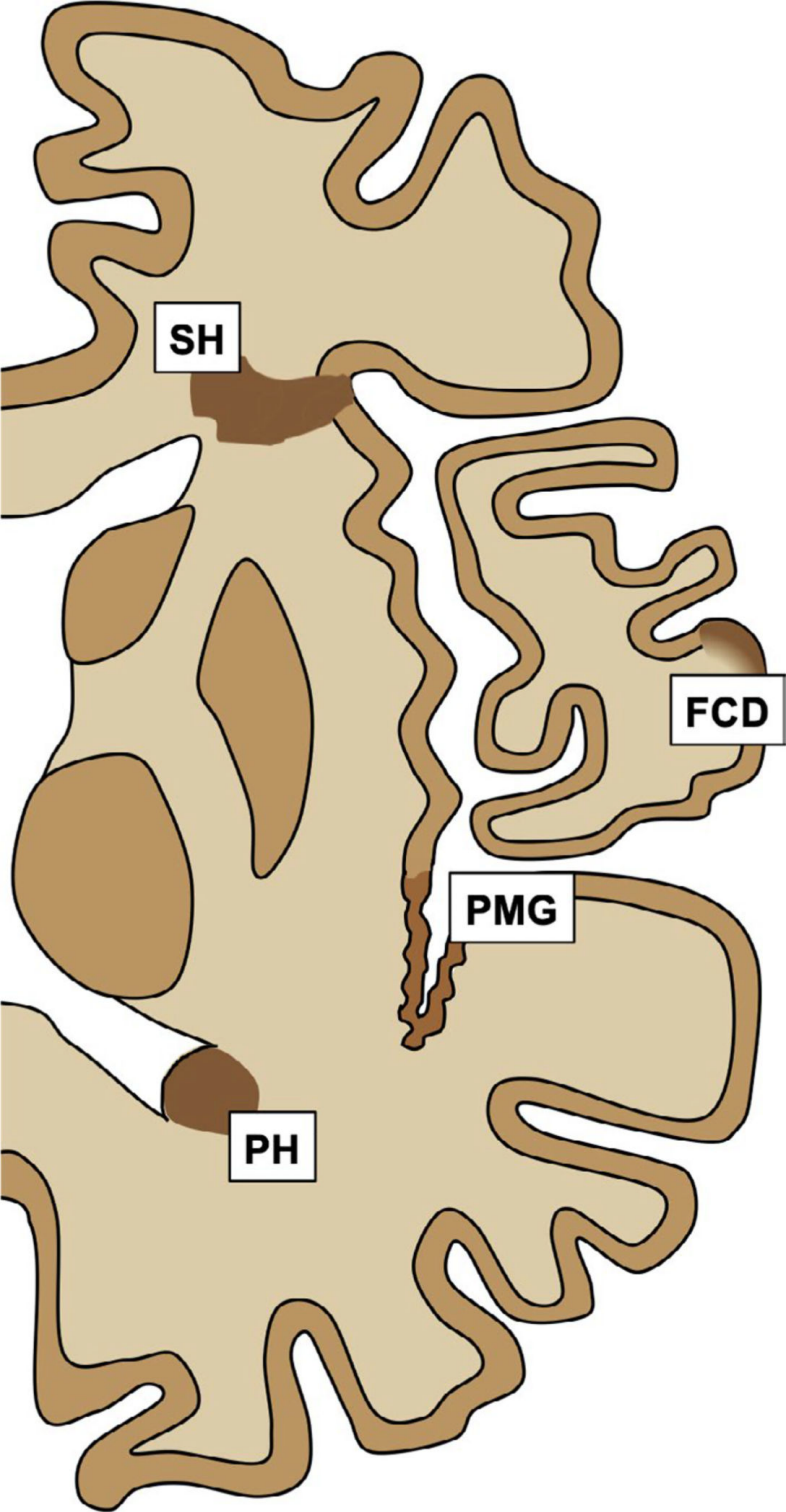
Typical manifestations of malformations of cortical development (MCD). On structural magnetic resonance imaging, periventricular heterotopia (PH) is characterized by cortical tissue misplaced near the ventricles, subcortical heterotopia (SH) by cortical tissue misplaced within deep white matter but often contiguous with the cerebral cortex, focal cortical dysplasia (FCD) by patches in the cerebral cortex with a blurred gray-white boundary, and polymicrogyria (PMG) by stretches of cerebral cortex with microscopic gyration. The color difference between MCD and normal cortex is for emphasis only

A total of 25 different lesions were delineated among the 13 patients (Table 1). The lesions represented each of the four different MCD types referred to in the introduction. An overview of typical manifestations for these MCD on structural MRI is shown in Figure 1. PH typically manifests as nodular masses of cortical tissue localized alongside the ventricular border or protruding into the ventricles. SH manifests as more irregular (curvilinear) patches of cortical tissue that are located mainly within deep white matter but that are often contiguous with the cerebral cortex. FCD resides within the cerebral cortex and has subtle manifestations, including a blurred gray-white boundary or apparent cortical thickening. Finally, PMG manifests as (often large) stretches of cerebral cortex with microscopic gyration.

### 3.2 | Comparison of MRI contrasts in normal brain tissue

**FIGURE 2:**
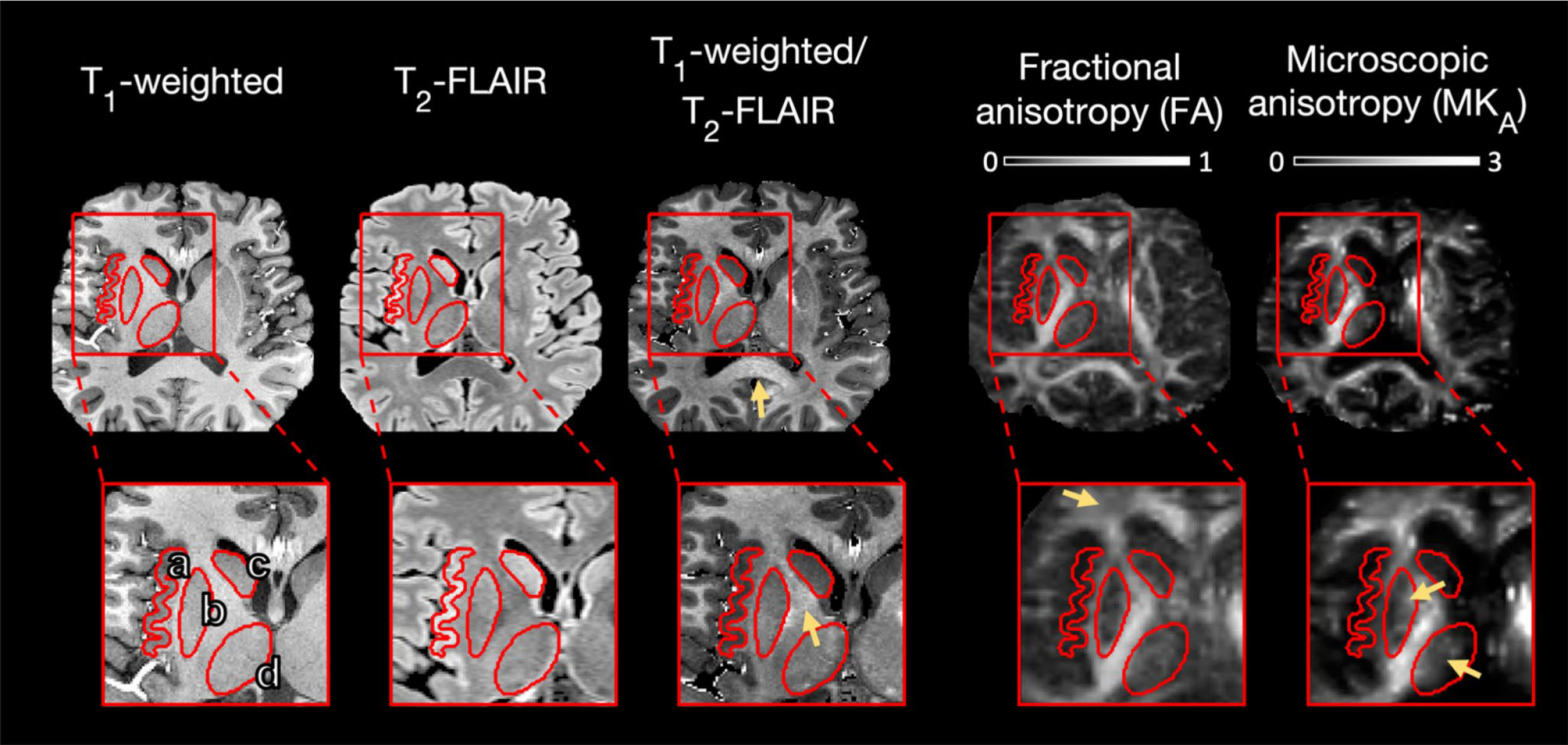
Imaging results from the normal brain. Magnified regions highlight different gray matter structures: the cerebral cortex (a), the putamen (b), the head of the caudate nucleus (c), and the thalamus (d). The T_1_-weighted image was darker in gray matter than in white matter, with lowest intensities in the cerebral cortex and in the head of the caudate nucleus followed by the putamen. The T_2_-fluid-attenuated inversion recovery (FLAIR) image featured a similar but opposite pattern. The image obtained as the ratio between the T_1_-weighted and the T_2_-FLAIR images featured a sharp contrast that emphasized myelin-rich regions such as the corpus callosum and the internal capsule (arrows). Both the fractional anisotropy (FA) map and the microscopic diffusion anisotropy (MK_A_) map were dark in gray matter and bright in white matter. However, the FA was comparatively low in the anterior corona radiata (arrow), and the MK_A_ revealed higher levels of microscopic anisotropy in the lateral thalamus and in the medial putamen (arrows). All contrasts were coregistered to the T_1_-weighted image space. The T_1_-weighted and T_2_-FLAIR intensities are in arbitrary units, and the FA and MK_A_ are dimensionless

Imaging results from the normal brain are exemplified in Figure 2. All MRI contrasts appeared to enable differentiation of normal cortex and white matter. The T_1_-weighted image was darker in gray matter (including both the cerebral cortex and deep gray matter) than in white matter, and the T_2_-FLAIR image featured the opposite pattern. Dividing the T_1_-weighted image with the (coregistered) T_2_-FLAIR image yielded a strong contrast that could plausibly reflect myelin, as suggested by Glasser et al,^34^ featuring the highest intensities in major white matter tracts such as the corpus callosum and the internal capsule. As for the dMRI contrasts, both the FA map and the MK_A_ map were dark in gray matter and bright in white matter, although they exhibited notable differences. The MK_A_ map was homogeneously bright in white matter, whereas the FA map featured darker areas in regions known to have crossing fibers. Conversely, the FA map was homogeneously dark in gray matter, whereas the MK_A_ map featured brighter gray matter regions, for example, in the lateral thalamus and in the medial putamen. The example illustrates the ambiguity in the FA due to the influence of orientation coherence. In white matter, variation in orientation coherence causes variation in FA even when diffusion anisotropy is uniformly high at the microscopic level (MK_A_). In gray matter, a low orientation coherence causes a uniformly low FA even when diffusion anisotropy varies at the microscopic level (MK_A_).

### 3.3 | Comparison of MRI contrasts in MCD

**FIGURE 3:**
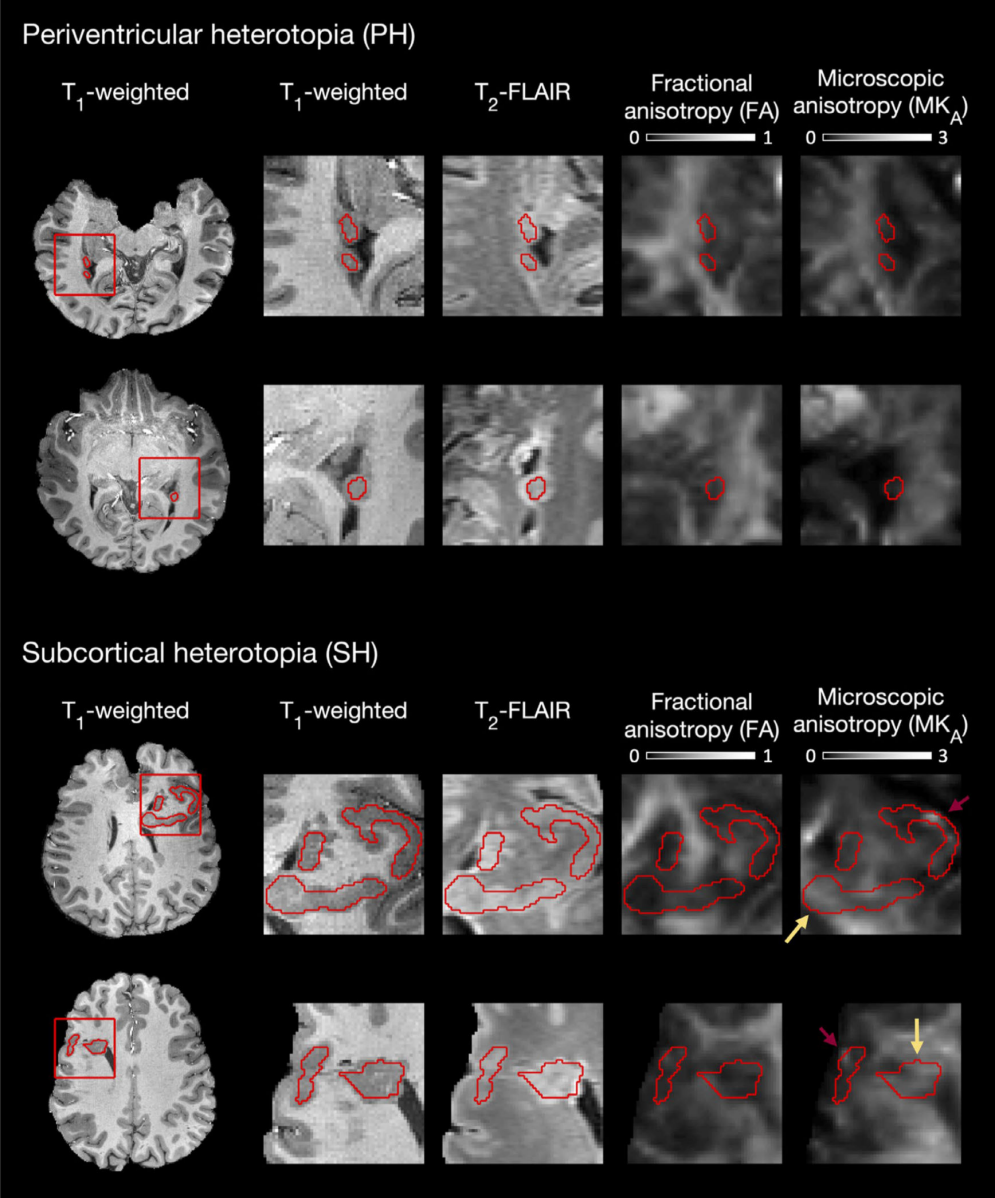
Imaging results from periventricular heterotopia (PH) and subcortical heterotopia (SH). In the T_1_-weighted, T_2_-fluid-attenuated inversion recovery (FLAIR), and fractional anisotropy (FA) contrasts, the PH and the SH appeared uniform, with intensities similar to nearby cortex. In the microscopic diffusion anisotropy (MK_A_) maps, the lesions were more heterogeneous. Dark and cortex-like MK_A_ was seen in the PH and in the parts of the SH that were contiguous with the cerebral cortex. Bright and white matter-like MK_A_ was seen in the parts of SH located within deep white matter (yellow arrows). The red arrows point out suspected sensitivity encoding-related artifacts in some voxels near the edge of the brain that resulted in an apparent high MK_A_. All contrasts were coregistered to the T_1_-weighted image space. The T_1_-weighted and T_2_-FLAIR intensities are in arbitrary units, and the FA and MK_A_ are dimensionless

**FIGURE 4:**
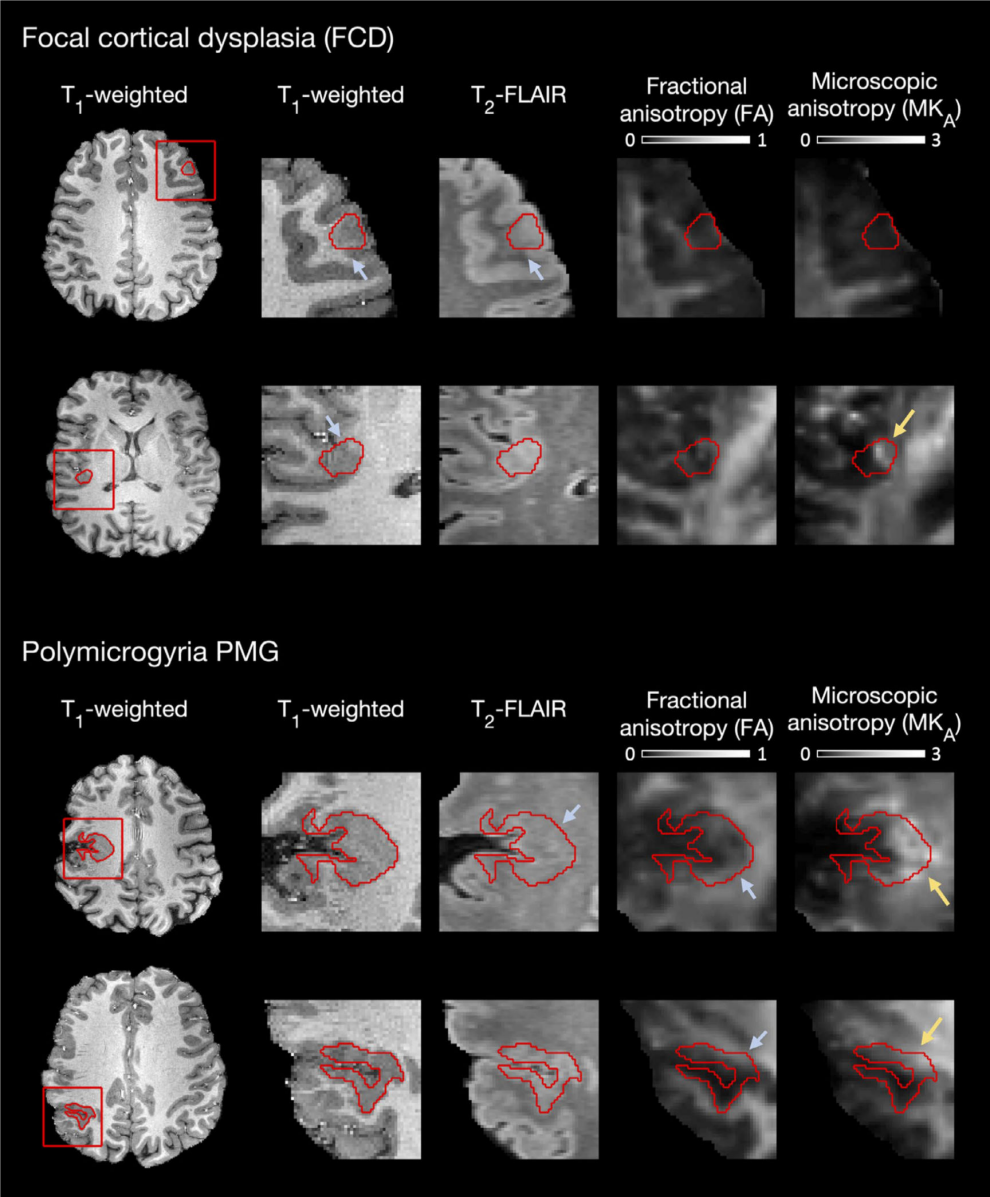
Imaging results from focal cortical dysplasia (FCD) and polymicrogyria (PMG). In the T_1_-weighted, T_2_-fluid-attenuated inversion recovery (FLAIR) and fractional anisotropy (FA) contrasts, the FCD and the PMG appeared uniform, with intensities similar to nearby cortex. Exceptions were blurred gray-white boundaries in FCD, a hypointense T_2_-FLAIR in one PMG where the T_1_-weighted image indicated apparent cortical thickening, and a bright FA close to the PMG borders (blue arrows). In the microscopic diffusion anisotropy (MK_A_) maps, both FCD and PMG exhibited mixed cortex-like and white matter-like parts. Dark and cortex-like MK_A_ was seen in most parts of FCD and in different parts of PMG, including the most superficial region in row 3 and the posterior gyrus in row 4. Bright and white matter-like MK_A_ was seen in the blurred gray-white boundary of some FCD, including in row 2, and in different parts of PMG, including the region of apparent cortical thickening in row 3 and the anterior gyrus in row 4 (yellow arrows). All contrasts were coregistered to the T_1_-weighted image space. The T_1_-weighted and T_2_-FLAIR intensities are in arbitrary units, and the FA and MK_A_ are dimensionless

Imaging results from the lesions are shown in Figure 3 (PH and SH) and Figure 4 (FCD and PMG). The figures show two sample lesions from each MCD type and are representative of the results from all 25 lesions in Table 1. In the T_1_-weighted images that were used to delineate the lesions, the lesions appeared uniformly cortex-like. Brighter areas with intensities similar to that of white matter were seen only in the blurred gray-white boundaries of the FCD (present in all FCD). The lesions appeared mainly cortex-like also in the T_2_-FLAIR images. However, white matter-like intensities were seen in some PMG (Table 1, lesions 11:1, 11:3, and 12:1), including a region where the T_1_-weighted image indicated severe cortical thickening (Figure 4, row 3). In the FA maps, all lesions were dark and similar to nearby cortex, although brighter areas were seen near the lesion border in two PMG lesions (Figure 4). In the MK_A_ maps, the lesions appeared more heterogeneous than in the other contrasts. In particular, the MK_A_ varied between dark and cortex-like to bright and white matter-like, both within and between different lesions. In PH, the MK_A_ was always cortex-like. In SH, the MK_A_ was cortex-like in the parts that were contiguous with the cerebral cortex but white matter-like in the parts located within deep white matter. These bright MK_A_ regions were seen in all but three of the eight SH lesions (where the extension into white matter was small or absent, Table 1, lesions 5:5, 6:2, and 4:4). In FCD, the MK_A_ was generally cortex-like, although white matter-like regions that overlapped the (T_1_-weighted) blurred gray-white boundaries were seen in two cases (Figure 4, row 2 and Table 1, lesion 7:1). The PMG lesions were the most heterogeneous in MK_A_. Two lesions were near exclusively cortex-like (Table 1, lesions 11:2 and 11:3), one lesion was near exclusively white matter-like (Table 1, lesion 12:1), and two lesions showed white matter-like parts next to cortex-like parts (Figure 4, rows 3–4), including the region of apparent cortical thickening.

**FIGURE 5:**
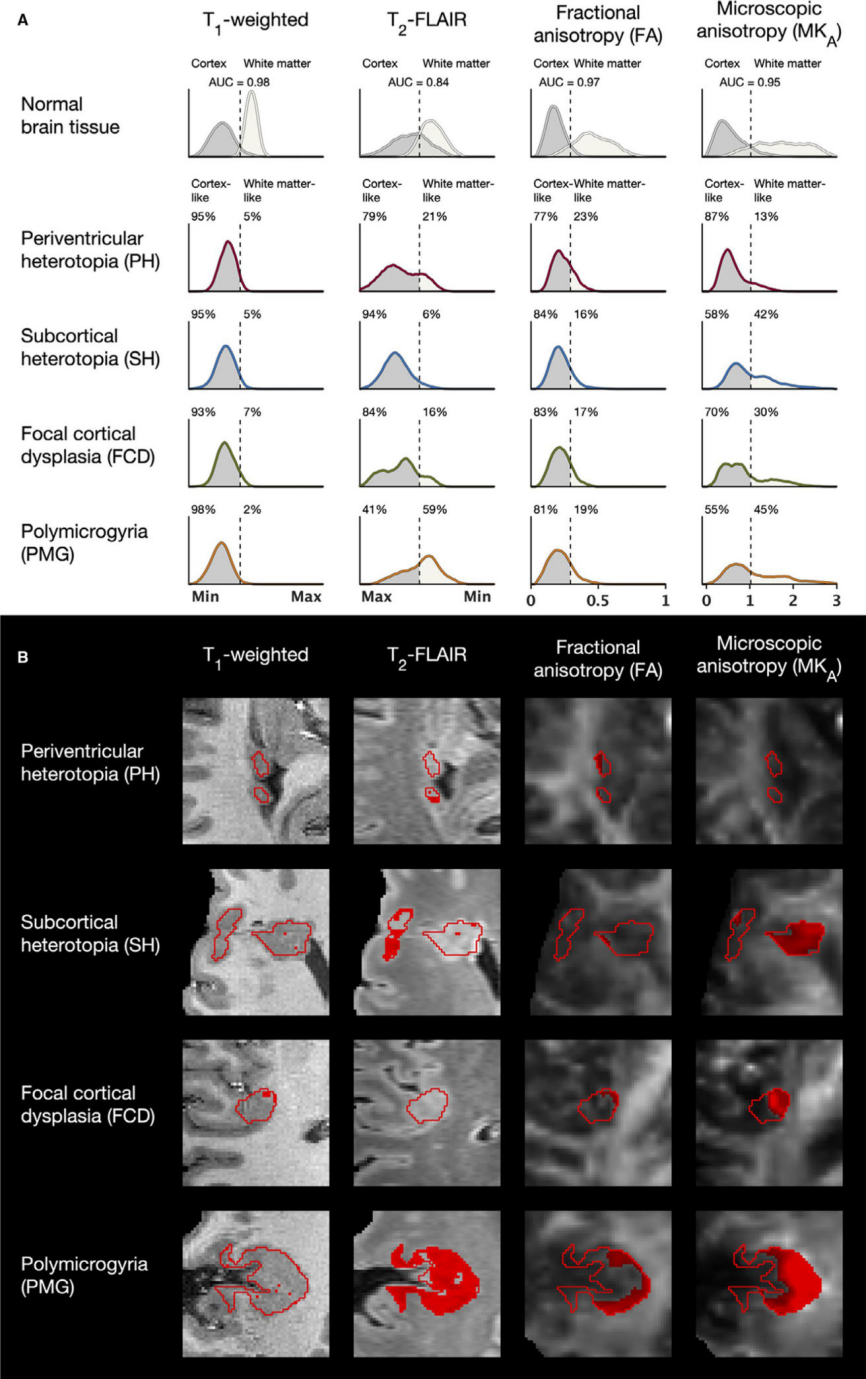
A, Intensity distributions in normal brain and in malformations of cortical development (MCD). In the T_1_-weighted, T_2_-fluid-attenuated inversion recovery (FLAIR), and fractional anisotropy (FA) contrasts, the MCD appeared primarily cortex-like, as indicated by the receiver operating characteristic (ROC)-derived thresholds for differentiation of normal cortex and white matter (dashed lines). The exceptions were that polymicrogyria (PMG) exhibited a large portion of white matter-like voxels in T_2_-FLAIR and that all MCD featured FA distributions with somewhat higher values than in the normal cortex. The microscopic diffusion anisotropy (MK_A_), however, revealed a mixed cortex-like and white matter-like content within all MCD except periventricular heterotopia (PH), with substantial portions of voxels exhibiting MK_A_ values far into the white matter range. The distributions were averaged across the regions of interest (ROIs) of normal cortex and white matter and all lesion ROIs of each MCD type. The area under the curve (AUC) indicates the area under the curve from the ROC analyses. T_1_-weighted and T_2_-FLAIR intensities are normalized, and FA and MK_A_ values are dimensionless. T_2_-FLAIR intensities are displayed from highest (Max) to lowest (Min). FCD, focal cortical dysplasia; SH, subcortical heterotopia. B, Classifications of tissue as white matter-like (red voxels) in one lesion of each MCD type. T_2_-FLAIR indicated very high portions of white matter-like voxels in some PMG. FA indicated white matter-like voxels near the border toward white matter of some lesions, whereas the MK_A_ indicated white matter-like voxels also in the deeper parts

Average intensity distributions in each contrast are shown in Figure 5A for normal brain tissue and for the different MCD types. The ROC-derived thresholds for differentiation of normal cortex and white matter (dashed lines) divide the MCD distributions between voxels classified as cortex-like and white matter-like (percentages indicated in the figure). Classifications of lesions from Figures 3 and 4 are shown in Figure 5B (white matter-like voxels in red), and the white matter-like percentage (WM%) for each individual lesion is listed in Table 1. In T_1_-weighted intensities, which were used for ROI definition, the overlap between normal cortex and white matter was minimal (Figure 5A, area under the curve [AUC] = 0.98) and the MCD distributions were primarily within the cortex range (WM% between 2% and 7%). For T_2_-FLAIR, the overlap between normal cortex and white matter was larger (AUC = 0.84). The distributions were primarily within the cortex range for PH, SH, and FCD (WM% between 6% and 21%). However, as exemplified in Figure 5B, T_2_-FLAIR indicated a substantial portion of white matter-like voxels in PMG (WM% = 59%), reflecting a high WM% in three of the five lesions (Table 1). For FA, the overlap between normal cortex and white matter was similar to that of T_1_-weighted intensities (AUC = 0.97). The range of FA values was markedly wider in white matter than in the cortex. All MCD types featured similar distributions that resembled that of the normal cortex but shifted toward slightly higher FA values. As a result, a portion of voxels within the white matter range of FA were present in all MCD (WM% between 16% and 23%) and were often found near the lesion borders (Figure 5B). For MK_A_, the overlap between normal cortex and white matter was small, although somewhat larger than in T_1_-weighted intensities and in FA (AUC = 0.95). Both cortex and white matter exhibited wider ranges of values in MK_A_ compared with the range of values in FA. The MCD distributions were considerably more heterogeneous in the MK_A_ than in the other contrasts. The distribution was primarily within the cortex range for PH (WM% = 13%). However, substantial portions of voxels far into the white matter range were seen in SH, FCD, and PMG (WM% between 30% and 45%).

## 4 | DISCUSSION

Tensor-valued dMRI yielded information on MCD independent from that obtained with conventional MRI contrasts. Lesions that appeared uniformly cortex-like in conventional MRI were revealed to feature white matter-like regions in maps of microscopic diffusion anisotropy (Figures 3–5, Table 1). Conventional MRI includes T_1_- and T_2_-weighted sequences, which are sensitive to myelin^5^ and iron,^11^ and FA from diffusion tensor imaging, which is sensitive to coherently ordered axons.^14,17^ The observed variation in microscopic anisotropy within and between MCD thus shows that some aspect of the microstructure can vary without affecting these conventional contrast mechanisms.

We argue that the observed variation in microscopic anisotropy within MCD is consistent with a variation in axonal content. In normal brain, microscopic anisotropy is low in cortex and high in white matter (Figure 2).^26–28,35,36^ This is consistent with axons but not dendrites as a cause of microscopic anisotropy, because the combined density of axons and dendrites (neurites) is similar in cortex and white matter.^28,37,38^ If axons and dendrites contributed equally to the microscopic diffusion anisotropy, the difference between cortex and white matter would be small. Even across normal gray matter regions, the variation in microscopic anisotropy appears related to a variation in axonal content.^28^ Here, higher MK_A_ was seen in the lateral thalamus, which is myelin-rich,^39^ and in the medial putamen, which is near the passage of striatopallidal fibers (Figure 2).^40^ In MCD, the observed variation in microscopic anisotropy is consistent with variations in axonal content expected from previous findings. For example, we found low MK_A_ in PH where the neural connectivity is limited,^41^ as well as in the dysplastic cortex of FCD and PMG where the neuronal density is reduced.^42,43^ Conversely, we found high MK_A_ in the white matter-embedded parts of SH, consistent with fiber-tracking findings of tracts passing through heterotopias located within white matter.^44^ We also found high MK_A_ in parts of FCD and PMG lesions located close to white matter (Figure 5B), which we interpret as subcortical white matter with myelin pathology, which can appear isointense with cortex in conventional MRI contrasts.^8^

Sensitivity to a voxel’s axonal content rather than its myelin content should allow a more robust differentiation of cortex and white matter. The T_1_-weighted and T_2_-weighted MRI contrasts provide a clear gray-white boundary in the normal adult brain.^5^ However, depending on the state of myelination within both cortical and noncortical tissue, the boundary may blur, exaggerate, or underestimate the cortex’s true extent.^6–8,10^ This complicates not only the clinical delineation of lesions but also the neuroscientific evaluation of cortical thickness, for example, during maturation.^7^ T_2_-weighted images may be less confounded by myelin content^8^ but may confound cortex and white matter also based on iron content. Here, T_2_-FLAIR indicated a high WM% in PMG similar to MK_A_ (Figure 5A). However, the two contrasts disagreed on individual lesions (for example, Table 1, lesions 11:3 and 13:1, and Figure 5B, row two), indicating that the T_2_-FLAIR finding represents a different effect. That effect could plausibly be iron content, seeing that the lesions with high WM% values were close to the motor and auditory regions (Table 1, lesions 11:1 and 12:1, and lesion 11:3, respectively), where iron concentrations are relatively high^45^ and where T_2_-weighted hypointensities have been demonstrated also in healthy subjects.^12,13^ The FA is sensitive to axonal content^14^ but, as illustrated in Figure 2, it provides a cloudy picture that is difficult to interpret by its dependence on orientation coherence. Here, the FA indicated higher white matter-like percentages in MCD than in normal cortex (Figure 5A) and FA maps were bright at the borders of high-MK_A_ areas in PMG (Figures 4 and 5B). However, the FA also showed tissue as homogeneous and dominantly cortex-like, where the MK_A_ showed considerable heterogeneity (Figures 3–5). As these conventional contrasts are unreliable in complex tissue with unknown myelin content, tensor-valued dMRI may become an important tool for tissue differentiation and for the evaluation of cortical thickness.

A more robust tool for differentiation of cortex and white matter would likely improve MCD delineation in presurgical evaluation of epilepsy. For surgery to achieve seizure freedom with minimal neurological sequelae, presurgical evaluation aims to delineate the “epileptogenic zone,” which is defined as the volume of cortical tissue that initiates or could initiate seizures.^45^ Recognizing tissue as white matter rather than cortex could result in a decrease of the planned resection volume and thereby a decreased risk of sequelae. For example, subcortical white matter may be spared in FCD and PMG (Figure 4).^8,10^ In large and often deep lesions such as SH (Figure 3), one may also prioritize the cortex-like parts for invasive investigations such as intracranial video-electroencephalographic recordings.^4^ Conversely, recognizing tissue as cortex rather than white matter could result in an increase of the planned resection volume and thereby an increased probability of seizure freedom. Examples of cortex resembling white matter in conventional MRI contrasts include cases with high myelin content,^7^ high iron content,^12,13^ and other unknown mechanisms.^6^

We identified four main limitations of this study. First, the gradient waveforms used to generate spherical b-tensors were not compensated for concomitant gradients, because the approach to generate compensated waveforms was not available at the start of this study. This is expected to bias the MK_A_.^47^ A positive bias was revealed by comparing the MK_A_ values obtained here, across the normal brain, with values obtained previously.^28^ A positive bias in MK_A_ has also been reported from previous data acquired with the 7-T MRI scanner used in the present study.^29^ However, the bias appeared spatially homogeneous and unlikely to have influenced the contrast, for example, between MCD and adjacent normal brain tissue. Second, we acknowledge that our results may be affected by partial volume effects. The dMRI data were affected by EPI-related geometric distortions, which are more pronounced on 7-T MRI,^29^ and the voxel size of 2 × 2 × 4 mm^3^ was large compared with the size of the cerebral cortex and some MCD. Although steps were taken to address this issue, partial volume effects may still have contributed to, for example, the relatively large percentage of high FA in some PH lesions (which are small, Table 1). Additionally, some voxels with suspected sensitivity encoding-related artifacts were observed within lesions close to the edge of the brain (Figure 3, red arrows). Our main finding of high-MK_A_ regions within MCD should be robust to these limitations, however, as these were present in many subjects and often large enough to involve multiple slices in the dMRI data’s native resolution. Third, microscopic anisotropy estimates may be biased by perturbations in T_2_-relaxation values between intra- and extracellular environments as, for example, in white matter lesions.^28^ Here, high MK_A_ was observed in the (T_1_-weighted) blurred gray-white boundaries of two of the four FCD lesions (Table 1). Both FCD lesions without high MK_A_ exhibited high mean diffusivity, which could indicate extracellular edema with long T_2_-relaxation values that dominate the signal from anisotropic structures.^28^ The importance of accounting for T_2_-relaxation as in, for example, our recent work,^26^ in the context of MCD should be explored in future studies. Fourth, the gradient waveforms used to generate linear and spherical b-tensors differed with respect to time-dependent diffusion encoding, which may positively bias the MK_A_.^48^ However, multiple studies have shown negligible diffusion time dependence in the human brain for the long encoding times used with clinical MRI scanners.^28,49,50^

In conclusion, mapping microscopic diffusion anisotropy revealed white matter-like regions within MCD that appeared cortex-like in conventional MRI. The results suggest that by reflecting axonal content rather than myelin content, microscopic anisotropy allows a more robust differentiation of cortex and white matter and improves MCD delineation. There are no technical barriers to performing tensor-valued dMRI. Techniques for mapping microscopic anisotropy are available at common clinical MRI systems^29^ and can be performed in just 3 minutes.^30^ Thus, tensor-valued dMRI has the potential to become an important tool for tissue differentiation in conditions that affect myelin and to benefit presurgical evaluation of epilepsy. Future studies should investigate the validity of microscopic anisotropy as a marker of axonal content in MCD. Such studies should address limitations of the current study and use larger sample sizes and correlations with histopathology. Future studies should also explore the technique’s potential for identifying “MRI-negative” lesions in drug-resistant epilepsy^3^ and compare its performance with a combination of conventional MRI contrasts, for example, through a machine learning approach.^51^

## Supplementary Material

Supplemental Material

## Figures and Tables

**TABLE 1:** Percentages of lesion voxels classified as white matter-like

	Lesion	Fig:row	ROI voxels, n	T_1_- weighted	T_2_- FLAIR	FA	MK_A_
PH	1:1	3:1	129	0	19	29	4
	1:2	—	145	0	94	79	83
	2:1	—	279	1	0	12	4
	2:2	—	243	8	2	2	2
	3:1	3:2	54	2	4	0	0
	4:1	—	160	1	49	4	10
	4:2	—	241	1	0	1	0
	4:3	—	52	6	2	58	0
		M (SD):	163 (86)	2 (3)	21 (34)	23 (30)	13 (28)
SH	5:1	—	492	6	0	2	86
	5:2	—	230	5	0	1	73
	5:3	3:3	2271	8	18	2	52
	5:4	—	2099	1	2	48	44
	5:5	—	788	2	0	31	7
	6:1	3:4	717	1	19	3	61
	6:2	—	497	1	2	20	11
	4:4	—	172	1	2	10	0
		M (SD):	908 (817)	3 (3)	5 (8)	15 (17)	42 (32)
FCD	7:1	—	235	12	no data	26	59
	8:1	4:1	488	2	45	5	1
	9:1	4:2	235	4	0	11	48
	10:1	—	184	4	0	15	7
		M (SD):	286 (137)	5 (4)	15 (26)	14 (9)	29 (29)
PMG	11:1	4:3	1684	2	92	25	53
	11:2	—	243	0	25	7	9
	11:3	—	643	0	94	8	13
	12:1	—	690	4	77	19	98
	13:1	4:4	834	0	7	23	48
		M (SD):	819 (531)	1 (2)	59 (40)	17 (9)	44 (36)

*Note.:* The classifications were based on the ROC-derived intensity thresholds for normal brain tissue (Figure 5). Gray-white blurring was observed for all FCD in T_1_-weighed and for lesion 8:1 in T_2_-FLAIR. Lesion, Patient (Table S1):lesion number; Fig:row, figure number (shown in):row number.

Abbreviations: FA, fractional anisotropy; FCD, focal cortical dysplasia; FLAIR, fluid-attenuated inversion recovery; M, mean; MK_A_, microscopic diffusion anisotropy; PH, periventricular heterotopia; PMG, polymicrogyria; ROI, region of interest; SD, standard deviation; SH, subcortical heterotopia.

## Key Points

- Delineation of MCD on MRI is central in presurgical evaluation of drug-resistant epilepsy
- Conventional T_1_- and T_2_-weighted contrasts may confound cortex with white matter when myelin content is altered
- The novel tensor-valued dMRI technique maps microscopic diffusion anisotropy, which is sensitive to axons rather than myelin
- Maps of microscopic anisotropy revealed white matter-like tissue in MCD that was cortex-like in conventional MRI contrasts
- Tensor-valued dMRI may improve differentiation of cortex and white matter and the delineation of MCD in presurgical evaluation of epilepsy
