# Supplementary material for: Tensor-valued diffusion MRI differentiates cortex and white matter in malformations of cortical development associated with epilepsy
Source: Epilepsia. Author manuscript; Available in PMC 2021 Aug 1. (PMC7963222; doi:10.1111/epi.16605)
Supplement: Supplemental Material [file NIHMS1674099-supplement-Supplemental_Material.pdf]

Supporting Tables

Table S1 Patient characteristics

Patient	Sex	Age
1	M	48
2	F	32
3	F	20
4	F	52
5	F	18
6	F	38
7	F	26
8	M	26
9	F	24
10	M	26
11	F	52
12	M	37
13	M	15

Table S2 Detailed parameters for the diffusion sequence

Scanner: Philips 7 Tesla Achieva	
Field strength [T]	7
Maximal gradient amplitude [mT/m]	64
Maximal gradient slew rate [T/m/s]	100
Head-coil	2Tx/32Rx-channel[a]
Imaging parameters	
Repetition time [ms]	3500
Echo time [ms]	89
Acquisition time [min:sec]	6:04
Parallel imaging factor (SENSE)	2
Partial Fourier	0.75
Bandwidth [Hz/pixel]	1933
Fat saturation	'strong'
Geometry	
Matrix size	112×112
Number of slices	15
Voxel dimensions [mm^3]	2×2×4
Diffusion parameters	
b_Δ-values	1 and 0[b]
b-values [ms/μm^2]	0,0.1,0.5,1.0,1.5,2.0[c]
Number of directions / b-value	1,6,6,10,11,16[d]
Number of samples	92
Maxwell compensation	No[e]
δ_1, δ_2, δ_P ($b_\Delta = 1$) [ms]	21.0, 21.0, 17.5
δ_1, δ_2, δ_P ($b_\Delta = 0$) [ms]	33.5, 25.5, 10.1

[a)]In Patient 8, a 1Tx/32Rx-channel setup was used, without dielectric pads.
[b)]The $b_\Delta = 0$ gradient waveform was optimized for minimal TE as in Sjölund et al[1].
[c)]All b-values were acquired for both b_Δ-values.
[d)]From Jones et al[2] and Leemans et al[3].
[e)]See Szczepankiewicz et al[4].
δ_1 and δ_2 and are the gradient waveform durations before and after the refocusing pulse with duration δ_P, respectively.

REFERENCES

1. Sjölund J, Szczepankiewicz F, Nilsson M, Topgaard D, Westin C-F, Knutsson H. Constrained optimization of gradient waveforms for generalized diffusion encoding. J Magn Reson. 2015;261:157-68.

2. Jones DK, Horsfield MA, Simmons A. Optimal strategies for measuring diffusion in anisotropic systems by magnetic resonance imaging. Mag Reson Med. 1999;42(3):515-25.

3. Leemans A, Jeurissen B, Sijbers J, Jones D. ExploreDTI: a graphical toolbox for processing, analyzing, and visualizing diffusion MR data. Proc Intl Soc Mag Reson Med. 2009.

4. Szczepankiewicz F, Westin CF, Nilsson M. Maxwell-compensated design of asymmetric gradient waveforms for tensor-valued diffusion encoding. Magn Reson Med. 2019;82(4):1424-37.
